# Prevalence and severity of bowel disorders in the third trimester of pregnancy

**DOI:** 10.1016/j.xagr.2023.100218

**Published:** 2023-05-17

**Authors:** Matteo Frigerio, Giuseppe Marino, Marta Barba, Stefania Palmieri, Alessandro Ferdinando Ruffolo, Rebecca Degliuomini, Pasquale Gallo, Giulia Magoga, Stefano Manodoro, Patrizia Vergani, Urogynecology-Pelvic Floor Working Group

**Affiliations:** 1 ASST Monza, San Gerardo Hospital, Monza, Italy (Dr Frigerio); 2 Urogynecology-Pelvic Floor Working Group, Monza, Italy (Drs Frigerio, Gallo, and Manodoro); 3 Department of Obstetrics and Gynecology, University of Milano-Bicocca, Milano, Italy (Drs Marino, Barba, and Palmieri); 4 San Raffaele University, Milano, Italy (Drs Ruffolo and Degliuomini); 5 San Giovanni di Dio Hospital, ASL Napoli 2 Nord, Frattamaggiore, Italy (Dr Gallo); 6 ULSS2 Marca Trevigiana, Oderzo Hospital, Oderzo, Italy (Dr Magoga); 7 ASST Santi Paolo e Carlo, San Paolo Hospital, Milano, Italy (Dr Manodoro); 8 Obstetric Division, Monza and Brianza Mother and Child Foundation, Monza, Italy (Dr Vergani).

**Keywords:** bowel disorders, incontinence, pelvic floor disorders, pregnancy, stypsis, third trimester

## Abstract

**BACKGROUND:**

Bowel-related disorders are common conditions associated with pregnancy and are a cause of significant distress and healthcare burden. However, there is a lack of data in the literature about these disorders.

**OBJECTIVE:**

This study aimed to investigate bowel dysfunctions during the third trimester of pregnancy in a large cohort of women using the validated bowel domain of the Italian version of the Pelvic Floor Questionnaire for Pregnant and Postpartum Women.

**STUDY DESIGN:**

This was a secondary analysis of a multicenter cross-sectional study conducted in hospitals in Italy and Italian-speaking Switzerland. Women in the third trimester of pregnancy were asked to complete the Italian Pelvic Floor Questionnaire for Pregnant and Postpartum Women.

**RESULTS:**

During the study period, 927 pregnant women in the third trimester of pregnancy responded to the questionnaire and were included in the analysis. Overall bowel dysfunctions were reported by 29.6% of patients. Constipation was reported by 66.6% of pregnant women, whereas symptoms of obstructed defecation were reported by 49.9% of patients. In contrast, urgency was reported by 41.1% of patients. Incontinence to flatus and incontinence to stool were reported by 45.1% and 2.8% of patients, respectively. Moreover, age >35 years, familiarity with pelvic floor disorders, nicotine abuse, and pelvic floor contraction inability were identified as independent risk factors for at least 1 bowel symptom.

**CONCLUSION:**

Bowel symptoms are extremely common in the third trimester of pregnancy and can greatly affect a patient's quality of life; therefore, bowel symptoms deserve to be investigated and managed properly. The use of validated questionnaires represents a precious tool to investigate functional symptoms that could be very frequent and disabling in this particular period of life for women.

**AJOG MFM at a Glance**

**Why was this study conducted?**

Bowel-related disorders are common conditions associated with pregnancy and are a cause of significant distress and healthcare burden, often underrated and underestimated in this fragile population.

**Key findings**

Bowel symptoms are extremely common in the third trimester of pregnancy and can greatly affect the patient's quality of life.

**What does this add to what is known?**

This study shows the prevalence of bowel disorders previously not reported or merely underreported and provides a base to ameliorate the management of patients with bowel disorders.

## Introduction

Pelvic floor disorders (PFDs) include a series of conditions usually related to lifestyle, hormonal, and obstetrical factors. These factors involve alterations in vaginal, bowel, lower urinary tract, and sexual functions, which are considered bothersome and negatively affect women's quality of life (QoL).^1^ In particular, bowel-related disorders, such as constipation and anal incontinence, are common conditions associated with pregnancy and are a cause of significant patient distress and healthcare burden. The etiopathogenesis of bowel disorders in pregnancy is believed to be multifactorial, involving changes in the hormonal environment, mechanical impairment of the growing uterus, dietary changes with a reduction in fiber intake, an increase in colonic water absorption, and a reduction in physical activity.^2^, ^3^, ^4^, ^5^, ^6^, ^7^ The available data on gastrointestinal (GI) dysfunction during pregnancy are extremely limited. Constipation seems to be second only to nausea as the most common GI complaint in pregnancy, with up to 40% of women suffering symptoms of constipation at some stage of pregnancy.^8^ There are some shreds of evidence that constipation may also represent a potential cause of PFD because of the damage of the pudendal nerve caused by excessive and prolonged straining, with subsequent impairment of the function of the pelvic floor musculature, which can lead subsequently to fecal or urinary incontinence, overactive bladder, or pelvic organ prolapse.^9^ Fecal incontinence is a much rarer dysfunction during pregnancy, with a prevalence of approximately 4%, but associated with a devastating effect on QoL and may also persist after childbirth.^10^ Once again, the etiopathogenesis seems to be multifactorial, and mechanisms other than delivery-related injuries to the anal sphincter and/or pudendal nerve play a role. Receptors for estrogens, androgens, and progesterone are expressed in the squamous epithelium of the anal canal, indicating that the hormonal environment can affect sphincter function. Similarly, the increased abdominal pressure of the pregnancy may induce conformational changes on the pelvic floor and lead to altered defecatory patterns.^11^^,^^12^ However, bowel dysfunctions other than constipation and fecal incontinence may affect pregnant women and determine the QoL impairment, including alteration in stool consistency, defecation frequency, urgency, soiling, flatus incontinence, and incomplete voiding.

To date, most studies are characterized by limited and heterogeneous populations and lack of validated instruments to evaluate bowel dysfunctions. In particular, until recently, no QoL questionnaire that is specifically designed and validated for pregnant women was available to investigate bowel disorders in this population. Validated QoL questionnaires are very important as they allow the assessment of the presence and severity of bowel symptoms and their effect on the patient's QoL, bringing to light conditions that may otherwise remain unrevealed. Recently, Metz et al^13^ have validated the German Pelvic Floor Questionnaire for Pregnant and Postpartum Women (PFQPP), which was subsequently validated in the Italian language by Palmieri et al.^14^ This questionnaire (Supplementary Material 1) includes an 11-item bowel domain, generating a score from 0 to 10 directly related to the total burden of bowel disorders. Moreover, it includes a domain for bowel dysfunction risk factor evaluation. This tool offers the opportunity to assess the severity, prevalence, risk factors, and effect of bowel symptoms on the QoL among pregnant Italian women, specifically in the third trimester of pregnancy.

This study aimed to investigate bowel dysfunctions during the third trimester of pregnancy in a large cohort of women using the validated bowel domain of the Italian PFQPP. Moreover, we aimed to investigate the effect of traditional risk factors for PFDs on bowel disorders in this particular population.

## Methods and Materials

This was a secondary analysis of a multicenter cross-sectional study that was conducted in 8 hospitals in Italy and Italian-speaking Switzerland.^11^ Women in the third trimester of pregnancy aged ≥18 years attending antenatal wards and outpatient clinics, from August 2020 to January 2021, were asked to complete the Italian PFQPP. The exclusion criteria were insufficient Italian language proficiency, age <18 years old, presence of diabetes mellitus, and presence of neurologic disorders. Questionnaire distribution was handled by the authors, and compilation was performed anonymously, to avoid embarrassment while answering the questions. The Italian PFQPP included an 11-item bowel domain, plus an additional domain for risk factors for PFDs. A complete English-translated version of the bowel domain is provided in Supplementary Material 2. A score of at least 1 point in each item was considered to be related to the presence of an alteration of the corresponding condition. Moreover, patients were asked to evaluate their bowel symptoms by answering the question “How much do your bowel symptoms bother you?” on a 5-answer Likert scale with the following choice of answers: “0, I do not have symptoms (not applicable)”; “1, not at all”; “2, a little”; “3, quite a lot”; and “4, very much.” Controls were defined as women answering “I do not have symptoms (not applicable)” or “not at all.” Apart from that, patients were defined as cases. The risk factor domain included the evaluation of the following as possible risk factors for bowel disorder: nicotine abuse, familiarity with PFDs, pelvic floor contraction (PFC) inability, BMI of >25 kg/m^2^, and age >35 years. Data are reported as mean±standard deviation for continuous variables and absolute (relative) frequency for noncontinuous variables. Continuous variables were analyzed using the Student *t* test for parametric variables and the Wilcoxon test for nonparametric variables, whereas the Pearson test was used for noncontinuous variables. The influence of risk factors on the specific symptoms was quantified with odds ratio (OR) with a 95% confidence interval (CI). All statistical analysis was performed using JMP (version 7.0, SAS Institute, Cary, NC). A *P* value of <.05 was considered significant. The study was approved by the Monza and Brianza's Mother and Child Foundation Ethics Committees (n 3116/2019).

## Results

During the study period, 927 patients responded to the questionnaire and were included in the analysis. Women were at 37 3/7 weeks of gestation at the time of questionnaire compilation, and 70.8% of the women were primiparous. Risk factor prevalence is shown in Table 1. In particular, 6% of women were nicotine users, 12.3% had familiarity with PFDs, and 3.1% considered themselves unable to contract pelvic floor muscles. All women who gave consent to participate in the study compiled at least part of the questionnaire (rate of missing items, 0.7%). Overall bowel dysfunctions (defined by answers “2, a little”; “3, quite a lot”; and “4, very much” to the question “How much do your bowel symptoms bother you?”) were reported by 29.6% of patients. The total bowel domain score was 1.7±1.2. Construct validity was confirmed; moreover, the total bowel domain score was significantly different between patients with and without symptoms (3.1±1.0 vs 1.2±0.7; *P*<.001), and the difference resulted in more than 1 point, which corresponds to the minimal important difference.^15^ Table 2 summarizes the prevalence of each specific bowel symptom in the population of the study. In our population, 66.6% of pregnant women reported constipation, with 72.7% reporting the need to strain. Abnormal consistency was reported by 67.9% of women, whereas symptoms of obstructed defecation were reported by 49.9% of patients. In contrast, urgency was reported by 41.1% of patients. Incontinence to flatus and incontinence to stool were reported by 45.1% and 2.8% of patients, respectively. Overall bowel symptoms were considered bothersome by 33.3% of women and negatively affected the QoL of 16.4% of the whole population. The influence of risk factors on bowel symptoms is shown in Table 3. Age >35 years was associated with strain (OR, 1.47; *P*=.018), urgency (OR, 1.40; *P*=.022), flatus incontinence (OR, 1.82; *P*=.001), other than QoL impairment (OR, 1.84; *P*<.001), and bothering symptoms (OR, 1.62; *P*=.001). Familiarity with PFD was identified as a risk factor for flatus incontinence (OR, 1.60; *P*=.019) and QoL impairment (OR, 2.07; *P*=.002). Nicotine abuse was associated with an increase in stool incontinence (OR, 2.99; *P*=.041), whereas PFC inability was associated with alterations in frequency (OR, 2.23; *P*=.033). Interestingly, a BMI of >25 kg/m^2^ has been identified as a protective factor against strain during defecation (OR, 0.69; *P*=.018). Moreover, nicotine abuse and age >35 years were found to be directly related to the global burden of bowel dysfunction, as expressed by the total domain score (*P*=.010 and *P*=.001, respectively) (Table 4).

**Table 1:** Population characteristics

Characteristics			Risk factor
Age (y)	33.0±4.9		>35 y 272 (29.8)
BMI (kg/m^2^)^a^	27.0±4.2		>25 kg/m^2^ 586 (64.4)
Nicotine	Never users 679 (73.6)	Previous users 189 (20.5)	Current users 55 (6.0)
Familiarity with PFDs	No 659 (71.3)	Not known 151 (16.3)	Yes 114 (12.3)
Pelvic floor contraction ability	Yes 629 (68.6)	Not known 260 (28.4)	No 28 (3.1)

Data are presented as mean±standard deviation for continuous variables and absolute number (percentage) for noncontinuous variables.

*BMI*, body mass index; *PFD*, pelvic floor disorder.

a At the time of questionnaire compilation.

**Table 2:** Prevalence and severity of bowel disorders

Variables	Prevalence	Score
1. Frequency	322 (35.1)	0.4±0.5
2. Consistency	624 (67.9)	0.7±0.5
3. Strain	668 (72.7)	1.0±0.8
4. Constipation	614 (66.6)	1.0±0.9
5. Flatus incontinence	416 (45.1)	0.6±0.8
6. Urgency	379 (41.1)	0.5±0.6
7. Stool smearing	57 (6.2)	0.1±0.3
8. Stool incontinence	26 (2.8)	0.0±0.2
9. Incomplete voiding	460 (49.9)	0.7±0.8
10. Quality of life impairment	151 (16.4)	0.2±0.5
11. Bothering symptoms	305 (33.3)	0.4±0.7

Mean severity score±standard deviation are provided (range from 0 to 3 [maximal severity]).

**Table 3:** Impact of risk factors

Variables	BMI>25 kg/m^2^	Age>35 y	Familiarity with PFDs	Nicotine abuse	PFC inability
1. Frequency	*P*=.074	*P*=.948	*P*=.699	*P*=.810	*P*=.033^a^ OR=2.23 (95% CI, 1.05–4.75)^a^
2. Consistency	*P*=.986	*P*=.652	*P*=.936	*P*=.088	*P*=.699
3. Strain	*P*=.018^a^ OR=0.69 (95% CI, 0.50–0.94)^a^	*P*=.024^a^ OR=1.47 (95% CI, 1.05–2.05)^a^	*P*=.520	*P*=.210	*P*=.577
4. Constipation	*P*=.296	*P*=.074	*P*=.497	*P*=.491	*P*=.056
5. Flatus incontinence	*P*=.983	*P*<.001^a^ OR=1.82 (95% CI, 1.36–2.42)^a^	*P*=.019^a^ OR=1.60 (95% CI, 1.08–2.39)^a^	*P*=.255	*P*=.510
6. Urgency	*P*=.631	*P*=.022^a^ OR=1.40 (95% CI, 1.05–1.86)^a^	*P*=.214	*P*=.506	*P*=.327
7. Stool smearing	*P*=.192	*P*=.231	*P*=.660	*P*=.136	*P*=.068
8. Stool incontinence	*P*=.556	*P*=.923	*P*=.913	*P*=.041^a^ OR=2.99 (95% CI, 1.00–8.99)^a^	*P*=.167
9. Incomplete voiding	*P*=.478	*P*=.685	*P*=.325	*P*=.482	*P*=.262
10. Quality of life impairment	*P*=.478	*P*<.001^a^ OR=1.84 (95% CI, 1.28–2.64)^a^	*P*=.002^a^ OR=2.07 (95% CI, 1.30–3.27)^a^	*P*=.123	*P*=.175
11. Bothering symptoms	*P*=.094	*P*=.001^a^ OR=1.62 (95% CI, 1.21–2.18)^a^	*P*=.133	*P*=.359	*P*=.423

*BMI*, body mass index; *CI*, confidence interval; *PFC*, pelvic floor contraction; *PFD*, pelvic floor disorder; *OR*, odds ratio.

a ORs with 95% CIs are provided for significant associations.

**Table 4:** Impact of risk factors on total bowel domain score

Variables	Presence of risk factor	Absence of risk factor	*P* value
BMI>25 kg/m^2^	1.7±1.2	1.8±1.2	.563
Age>35 y	1.9±1.2	1.7±1.2	.001^a^
Familiarity for PFDs	1.9±1.1	1.7±1.2	.179
Nicotine abuse	2.2±1.4	1.7±1.2	.010^a^
PFC inability	1.9±1.4	1.7±1.2	.502

Data are presented as mean±standard deviation.

*BMI*, body mass index; *PFC*, pelvic floor contraction; *PFD*, pelvic floor disorder.

a Statistically significant correlation.

## Comment

### Principal findings

Bowel symptoms, such as constipation and fecal incontinence, are common during pregnancy and are a cause of significant patient distress and healthcare burden. However, bowel function in the context of pregnancy is an underinvestigated topic by care providers. The real prevalence of bowel dysfunctions in this population is not well defined because of the risk of underreporting and the lack of tools validating bowel disorders in this particular population of women. Moreover, common criteria used in the general population to define constipation, such as Rome IV criteria, are not validated in the pregnant population.^16^ Here, we aimed to evaluate, in the third trimester of pregnancy, the prevalence, severity, risk factors, and effect of bowel disorders on the emotional well-being of pregnant women using a validated questionnaire for pregnant women (Italian PFQPP). We found the following:

• Overall bowel dysfunctions were reported by 29.6% of patients.
• Abnormal consistency and obstructed defecation were reported by 67.9% and 49.9% of patients, respectively.
• Urgency, incontinence to flatus, and incontinence to stool were reported by 41.1%, 45.1%, and 2.8% of patients, respectively.
• Bowel symptoms were considered bothersome by 33.3% of women and negatively affected the QoL of 16.4% of the population.
• Familiarity with PFD, nicotine abuse, PFC inability, and age >35 years were found to be associated with at least 1 symptom or score.

### Results in the context of what is known

In our study, we found that 66.6% of pregnant women in the third trimester of pregnancy complained of constipation, with 67.9% of patients reporting the need for strain. Traditionally, it is considered that the prevalence of constipation increases in the first and second trimesters of pregnancy and decreases in the third trimester of pregnancy and in the postpartum period.^17^, ^18^, ^19^ This is believed to be related to hormonal factors with progesterone playing a pivotal role in bowel down-regulation.^2^^,^^3^ However, other factors, such as the mechanical changes associated with advancing gestation and fetal growth, may represent major influences. Interestingly, in our study, this prevalence was higher than previously reported by other authors in the same period of pregnancy, ranging from 16% to 36%.^17^, ^18^, ^19^ However, considered studies evaluated the prevalence of constipation in pregnancy based on the Rome criteria, which has never been validated in a pregnant population. Moreover, it tends to underestimate the condition, as shown in a study by Ponce et al,^20^ where the prevalence of constipation in fertile-age women based on self-report was 2-fold higher than that based on the Rome criteria. Interestingly, when constipation is self-reported instead of applying the Rome criteria in the same population of women pregnant during the last trimester, the prevalence rose from 36% to 55%, which is much more similar to the rate in our survey.^19^

Fecal incontinence is associated with fewer problems in pregnancy. However, its emotional, physical, social, and mental effects might be devastating.^21^, ^22^, ^23^ Moreover, its prevalence is likely underestimated, as demonstrated by the finding that only 14% of symptomatic women sought medical attention.^24^ The maintenance of fecal continence is a complex system involving normal stool consistency and volume, normal colonic transit time, a compliant rectum, innervation of the pelvic floor and anal sphincters, and the interplay among the puborectalis muscle, rectum, internal anal sphincter, and external anal sphincter. Although most published studies focus on the way of delivery as a risk factor for incontinence, there is a lack of data on the prevalence of this condition in pregnancy.^25^^,^^26^ Our data show a 41.4% of urgency in the third trimester of pregnancy, with 45.1% of flatus incontinence and 2.8% of stool incontinence, showing a problem far from being unusual. Although several studies have analyzed the prevalence of anal incontinence after pregnancy, there are limited data on the characteristics of this problem during this period. In late pregnancy, these studies have shown a prevalence ranging between 3% and 10% of fecal incontinence, affecting the QoL.^10^^,^^27^ In a large cross-sectional study, the prevalence of anal incontinence, intended as flatus or stool loss, was higher during late pregnancy, with an important effect on QoL.^28^ In particular, 40.8% of the population analyzed showed at least 1 episode of anal incontinence, which is consistent with our rate of flatus incontinence. Interestingly, anal incontinence–related symptoms seem to improve in the late postpartum period and after 12 to 36 months, according to available reports.^29^^,^^30^

### Clinical implications

Our study evaluated the role of risk factors in the development of bowel disorders with age >35 years, familiarity with PFDs, nicotine abuse, and PFC inability identified as independent risk factors for at least 1 bowel symptom. Specifically, age >35 years was associated with a greater number of symptoms, including the need for strain, urgency, flatus incontinence, and overall bothersome bowel disorders. Moreover, both age >35 years and familiarity with PFDs were associated with significant QoL impairment (OR: 2.07 and 1.84, respectively). Both of them have been previously identified as risk factors for PFDs in pregnant and postpartum women.^11^ In addition, nicotine abuse was associated with stool incontinence (OR, 2.99; *P*=.041). This has been previously observed by other authors in the female population.^31^ Nicotine is thought to have a defecation stimulus. In particular, it allows the release of nitric oxide, a molecule that has shown a smooth muscle relaxing effect.^32^ We can hypothesize that the association between the relaxation of bowel smooth muscle and the augmentation of abdominal pressure because of the pregnant uterus can be a cause of this major risk of stool incontinence.

### Research implications

All these associations, previously not reported or just considered of little account, could be used as a base for better counseling in the pregnant population, trying to change the modifiable attitudes and teaching high-risk patients better behavior rules not only to treat disorders but also to prevent disorders.

### Strength and limitations

The major strengths of our study are the large and homogeneous population included, the multicenter design, and the use of a specific validated questionnaire, which allowed the evaluation of symptom prevalence, severity, and associated risk factors. A study limitation is represented by anonymity, which makes it impossible to evaluate bowel function before the onset of pregnancy, symptoms trend after birth, and, in general, all the other potential influencers of bowel function not investigated by the questionnaire itself. However, anonymous questionnaires are more likely to uncover symptoms that might otherwise remain occult, and this may be particularly important for symptoms that could be difficult to refer to physicians because of embarrassment.

## Conclusion

Our study proves that bowel symptoms are extremely common in the third trimester of pregnancy and can greatly affect a patient's QoL; therefore, bowel symptoms deserve to be investigated and managed by caregivers. Here, more than half of the women suffered from at least 1 bowel disorder, with constipation and strain being the most widely reported. Interestingly, 2.8% of pregnant women in the last trimester of pregnancy suffered from stool incontinence. Age, familiarity with PFDs, nicotine abuse, and PFC inability were identified as significant risk factors for at least 1 bowel symptom, with the first 2 items also being a predictor of significant QoL impairment. The use of validated questionnaires represents a precious tool to investigate functional symptoms that could be very frequent and disabling in pregnancy. This creates the opportunity to improve prevention, early diagnosis, and treatment, improving pregnant women's QoL.

## Appendix Supplementary materials

Image, application 1

Image, application 2

## Footnotes

Supplementary material associated with this article can be found in the online version at doi:10.1016/j.xagr.2023.100218.
