# Supplementary material for: Prevalence and severity of bowel disorders in the third trimester of pregnancy
Source: AJOG Glob Rep. 2023 May 17;3(3):100218. doi: 10.1016/j.xagr.2023.100218 (PMC10461240; doi:10.1016/j.xagr.2023.100218)
Supplement: Supplementary file 2 [file mmc2.pdf]

Questionario pavimento pelvico
per gestanti e postpartum

Modulo fattori di rischio **Rischio**

Altezza<br>☐☐☐ cm	Peso<br>☐☐ kg	Peso pregravidico<br>per BMI ☐☐ kg	BMI<br>☐☐,☐	☐ >25
Età		☐☐ anni		☐ >35
In famiglia ci sono/sono state donne con incontinenza urinaria,intestinale, prolasso degli organi pelvici?		☐ no	☐ non so	☐ si
Fuma?		☐ no	☐ ho smesso	☐ si
Ritiene di essere in grado di contrarre la muscolatura del pavimento pelvico?		☐ si	☐ non so	☐ no

Funzionalità della vescica

1. ogni quanto urina nell'arco della giornata?	0. ogni 3 ore	1. ogni 2 ore	2. ogni ora	3. più spesso
2. quante volte si sveglia la notte per urinare ?	0. 0-1 volta	1. 2 volte	2. 3 volte	3. più di 3 volte
3. ha perdite urinarie mentre dorme?	0. mai	1. talvolta/meno di una volta la settimana	2. spesso/una o più volte la settimana	3. molto spesso/quotidianamente
4. lo stimolo urinario è così forte da dover andare subito in bagno?	0. mai	1. talvolta/meno di una volta la settimana	2. spesso/una o più volte la settimana	3. molto spesso/ quotidianamente
5. quando sente l'urgenza di urinare, perde urina prima di arrivare in bagno?	0. mai	1. talvolta/meno di una volta la settimana	2. spesso/una o più volte la settimana	3. molto spesso/ quotidianamente
6. perde urina quando tossisce, starnutisce, ride, fa sforzi o fa sport?	0. mai	1. talvolta/meno di una volta la settimana	2. spesso/una o più volte la settimana	3. molto spesso/ quotidianamente
7. Il flusso urinario è debole, rallentato o prolungato?	0. mai	1. talvolta/meno di una volta la settimana	2. spesso/una o più volte la settimana	3. molto spesso/ quotidianamente
8. ha la corretta percezione di quanto sia piena la vescica?	0.si/ sempre	1. quasi sempre	2.talvolta	3.no/mai
9. ha la sensazione di non svuotare completamente la vescica?	0. mai	1. talvolta/meno di una volta la settimana	2. spesso/una o più volte la settimana	3. molto spesso/ quotidianamente
10. deve spingere per poter urinare?	0. mai	1. talvolta/meno di una volta la settimana	2. spesso/una o più volte la settimana	3. molto spesso/ quotidianamente
11. usa salvaslip o assorbenti a causa delle perdite urinarie?	0.mai	1.talvolta/solo come precauzione	2.spesso/durante attività fisica o quando sono raffreddata	3. molto spesso/ quotidianamente
12. limita l'assunzione di liquidi per evitare le perdite urinarie?	0. mai	1. talvolta/meno di una volta la settimana	2. spesso/una o più volte la settimana	3. molto spesso/quotidianamente

13. quando urina ha bruciore, fastidio o dolore?		0. mai	1. talvolta/meno di una volta la settimana	2. spesso/una o più volte la settimana	3. molto spesso/ quotidianamente
14. quanto spesso ha infezioni alle vie urinarie?		0. raramente	1. 1-3 volte all'anno	2. 4-12 volte all'anno	3. una o più volte al mese
15. le perdite urinarie influiscono sulla sua vita quotidiana (ad es. su attività fisica, lavoro, spesa, uscite)?	☐ non mi riguarda, perché non ho sintomi	0.per niente	1.un poco	2.abbastanza	3.molto
16. Quanto la infastidiscono questi disturbi alla vescica?	☐ non mi riguarda, perché non ho sintomi	0.per niente	1.un poco	2.abbastanza	3.molto

Funzionalità intestinale

1. quante volte si scarica?	0. da ogni 3 giorni a quotidiana mente	1.più di una volta al giorno	1.ogni 4 giorni o più raramente	2.meno di una volta alla settimana
2. normalmente com'è la consistenza delle feci?	0. morbide/ composte	1.varia	1.molto dure	2.liquido/ pastose
3. spinge molto per scaricarsi?	0. mai	1. talvolta/meno di una volta la settimana	2. spesso/una o più volte la settimana	3. molto spesso/ quotidianamente
4. soffre di stitichezza?	0. mai	1. talvolta/meno di una volta la settimana	2. spesso/una o più volte la settimana	3. molto spesso/ quotidianamente
5. soffre di flatulenza e non riesce a trattenere l'aria?	0. mai	1. talvolta/meno di una volta la settimana	2. spesso/una o più volte la settimana	3. molto spesso/ quotidianamente
6. sente improvvisamente l'urgenza di evacuare?	0. mai	1. talvolta/meno di una volta la settimana	2. spesso/una o più volte la settimana	3. molto spesso/ quotidianamente
7. Trova gll slip o l'assorbente sporchi di feci?	0. mai	1. talvolta/meno di una volta la settimana	2. spesso/una o più volte la settimana	3. molto spesso/ quotidianamente
8. ha perdite di feci o non riesce a trattenerle?	0. mai	1. talvolta/meno di una volta la settimana	2. spesso/una o più volte la settimana	3. molto spesso/ quotidianamente
9. ha la sensazione di non riuscire a svuotare completamente l'intestino?	0. mai	1. talvolta/meno di una volta la settimana	2. spesso/una o più volte la settimana	3. molto spesso/ quotidianamente

10. questi sintomi influiscono sulla sua vita quotidiana? (impegni quotidiani, sport, lavoro, spesa, uscite)?	☐ non mi riguarda, perché non ho sintomi	0.per niente	1.un poco	2.abbastanza	3.molto
11. quanto la infastidiscono i problemi intestinali?	☐ non mi riguarda, perché non ho sintomi	0.per niente	1.un poco	2.abbastanza	3.molto

Prolasso

1. Ha la sensazione di avere un corpo estraneo in vagina?		0. mai	1. talvolta/meno di una volta la settimana	2. spesso/una o più volte la settimana	3. molto spesso/ quotidianamente
2. Percepisce un abbassamento dell'utero o della vagina?		0. mai	1. talvolta/meno di una volta la settimana	2. spesso/una o più volte la settimana	3. molto spesso/ quotidianamente
3. Mentre solleva pesi, cammina, corre, le sembra che vagina o utero si abbassino?		0.per niente	1.un poco	2.abbastanza	3.molto
4. Questi disturbi quanto influiscono sulla sua vita quotidiana?	☐ non mi riguarda, perché non ho sintomi	0.per niente	1.un poco	2.abbastanza	3.molto
5. Quanto la disturba il prolasso?	☐ non mi riguarda, perché non ho sintomi	0.per niente	1.un poco	2.abbastanza	3.molto

Sessualità

E' sessualmente attiva?	☐ no	☐ raramente	☐ regolarmente	
Nel caso non abbia rapporti, per quale motivo?	☐ Non ho un partner	☐ Il partner ha problemi/ impotenza	☐ Non ho alcun desiderio	☐ I rapporti sessuali sono spiacevoli perché………… …………………
Ha avuto esperienze sessuali che l'hanno traumatizzata?	☐ no			☐ si
1. Durante i rapporti sessuali, la vagina è sufficientemente lubrificata?	☐ si			☐ no
2. Qual è il livello di sensibilità durante il rapporto?	0.elevato	1.discreto/ scarso	2 .nullo	3. ho dolore
3. Pensa che la sua vagina sia ridondante o lassa?	0. no/mai	1. talvolta	2. spesso	3. sempre
4. Pensa che la sua vagina si stretta o rigida?	0. no/mai	1. talvolta	2. spesso	3. sempre

5. Ha dolori durante il rapporto?		0. no/mai	1. talvolta	2. spesso	3. sempre
6. Se ha dolore durante il rapporto, dove?		1. alla penetrazione		2. in profondità/ nella pelvi	3. entrambi
7. Durante il rapporto, ha perdite incontrollate di urina o feci?		0. no/mai	1. talvolta	2. spesso	3. sempre
8. Questi sintomi influenzano la sua vita sessuale?	□ non mi riguarda, perché non ho sintomi	0.per niente	1.un poco	2.abbastanza	3.molto
9. Quanto la infastidiscono questi sintomi?	□ non mi riguarda, perché non ho sintomi	0.per niente	1.un poco	2.abbastanza	3.molto

Score (sezione da non compilare)

**Funzionalità della vescica**	domande da 1 a 16	score ☐☐/48=	☐,☐	x10=	☐☐
**Funzionalità intestinale**	domande da 1 a 11	score ☐☐/31=	☐,☐	x10=	☐☐
**Prolasso**	domande da 1 a 5	score ☐☐/15=	☐,☐	x10=	☐☐
**Sessualità**	domande da 1 a 9	score ☐☐/24=	☐,☐	x10=	☐☐
Score vescica+ score intestino+ score prolasso+ score attività sessuale=					

Modulo postpartum **Rischio**

Quanti figli ha partorito?	☐☐	Durante quanti parti è stata usata la ventosa?			☐
Quanti parti sono avvenuti tramite taglio cesareo?	☐	Durante quanti parti è stato usato il forcipe?			☐
Quanto pesava alla nascita il figlio più grosso?				☐☐☐☐ g	☐ >4000g
Durante uno dei parti, ha avuto danni a carico dello sfintere anale o dell'intestino				☐ no	☐ si
Dopo il parto ha avuto dolori vaginali, perineali o intestinali?				☐ no	☐ si
Dopo aver partorito, pensa di aver rielaborato i dolori del parto?		☐ si	☐ in gran parte	☐ un poco	☐ no
Pensa di aver rielaborato le paure che aveva durante il parto?		☐ si	☐ in gran parte	☐ un poco	☐ no
